# Comparison of three commercial serological tests for the detection of *Chlamydia abortus* infection in ewes

**DOI:** 10.1186/s13620-018-0124-2

**Published:** 2018-05-29

**Authors:** L. M. O’Neill, Á. O’Driscoll, B. Markey

**Affiliations:** School of Veterinary Medicine, University College Dublin, Room 07A, Belfield, Dublin 4, Ireland

**Keywords:** *Chlamydia abortus*, Enzootic abortion of ewes, ELISA sensitivity, ELISA comparison

## Abstract

**Background:**

20.9% of diagnosable abortions in Ireland in 2015 were caused by *Chlamydia abortus* infection. Abortion usually occurs in the last 2–3 weeks of gestation, and up to 30% of ewes may be affected in naïve flocks. Serological diagnosis of EAE in flocks using LPS or whole bacteria as antigens is often hindered by cross reactions with *C. pecorum.* Although the complement fixation test is the official test for diagnosis of EAE, more sensitive and specific ELISA based tests have been developed. This study aimed to compare three commercial ELISA kits to detect *C. abortus* antibodies in ewes and to determine which of the kits had the highest sensitivity. The IDvet kit utilises a MOMP peptide antigen, the MVD-Enfer kit is based on a POMP90–3 antigen while the LSI kit plates are coated with chlamydial LPS. The study also aimed to examine the potential of these ELISAs to distinguish infected animals that go on to abort compared to those that have live lambs. Ewes were vaccinated with either a commercial live vaccine (*n* = 10) or Tris-buffer sham inoculation (*n* = 9) 5 months prior to gestation, these ewes were then challenged with *C. abortus* (1 × 10^6^ IFU/ml) on day 90 of gestation. Sera were collected at pre-vaccination, 14 days post vaccination, 35 days post vaccination, pre-challenge, 35 days post challenge and 3 weeks post lambing/abortion (~ 70 days post challenge) and tested using the 3 aforementioned ELISAs to determine if one ELISA was more sensitive at detecting circulating anti-chlamydial antibodies.

**Results:**

Sensitivity was highest with the LSI test kit at 94.74%, followed by the MVD-Enfer and IDvet kits, at 78.95 and 73.68% respectively. Ewes vaccinated with Enzovax became seropositive at 14 days post vaccination with all kits. Following challenge at day 90 of gestation, antibody titres steadily rose in all groups of ewes. With all ELISA kits, antibody levels were higher in ewes that aborted compared to ewes that had live lambs at 35 days post challenge and three weeks post lambing, and statistically significantly higher antibody levels were recorded in ewes that aborted compared to ewes that had live lambs using the MVD-ENFER ELISA at three weeks post lambing (*P* = 0.0482).

**Conclusions:**

The LSI assay was the most sensitive out of the three kits tested in this study, when sera were tested at three weeks post lambing. As the LPS used in this kit is cross-reactive with all chlamydia, it is good for identifying flocks infected with any chlamydial species, but it is not considered specific for *C. abortus*. Furthermore, antibody levels were higher in ewes that aborted compared to ewes that had live lambs, at both 35 days post challenge and at three weeks post lambing. Future work should include evaluation of a larger number of sera at a wider range of time-points as well as an estimation of the specificity of commercially available assays.

## Background

Enzootic abortion of ewes (EAE) is the second most common infectious cause of ovine abortion in Ireland, causing 20.9% of diagnosable abortions in 2015 [1]. The causative agent, *Chlamydia abortus,* is a serious zoonotic pathogen in pregnant women which makes accurate testing essential in the diagnosis and control of infected sheep [2]. The disease manifests itself as a placentitis, leading to abortion in the last 2 to 3 weeks of gestation [3]. Aborting ewes are the principal means of transmission of infection but rarely show prior clinical signs. As a result early, detection of infection during pregnancy is vital in permitting therapeutic intervention and controlling the spread of disease.

Serological diagnosis of EAE in flocks is challenging for a number of reasons. When serological tests such as the complement fixation test (CFT) use LPS or whole bacteria as antigens in the diagnosis of *C. abortus* infection, specificity tends to be low as cross reactions with *Chlamydia pecorum* are often observed [4]. *Chlamydia pecorum* mainly causes inapparent enteric infections [5], but is also known to cause arthritis, conjunctivitis and pneumonia [3]. Cross-reactive antibodies may arise from other chlamydial species and certain Gram-negative bacteria such as *Acinetobacter* spp. [4]. Thus, specific diagnostic tests are required to properly understand the epidemiology of EAE and in implementing control strategies. The diagnosis of EAE is further complicated by the ability of *C. abortus* to cause latent infections [6]. Pathological lesions in the ovine placenta do not usually develop until day 90 of gestation [7].

The CFT is the most widely used technique for serological diagnosis of EAE and is considered the gold standard test for official trade purposes [8]. However, advances have been made in the development of more sensitive and specific tests such as ELISAs that specifically detect antibodies to a range of chlamydial antigens including major outer membrane protein (MOMP) and polymorphic outer membrane proteins (POMP) [9].

The aim of this study was to compare three commercial ELISAs designed to detect antibodies to *C. abortus* in ewes. Each ELISA utilises a different chlamydial antigen. In the IDvet kit, wells are coated with a MOMP peptide antigen, which is known to have genus-, species-, subspecies- and serotype-specific immunodomains [10]*.* Wells of the MVD-Enfer kit are coated with a POMP90–3 antigen considered to be specific for *C. abortus* [11]. The LSI kit uses chlamydial LPS as the antigen coating the wells. This is a genus-specific antigen common to all chlamydial species [12].

The study aimed to determine which of the three kits had the highest sensitivity and to evaluate the influence of the different antigens used in the assays on test characteristics. Furthermore, the study aimed to evaluate the potential of the different kits to identify ewes that subsequently progress to abortion.

## Method

Sera from two groups of ewes (*n* = 19) were tested using three different commercial ELISA kits: IDvet, LSI and MVD-Enfer. The sera were generated as part of a vaccination trial [13]. This trial involved 5 different treatment groups including the testing of novel recombinant protein vaccines against EAE. The sera from the positive control ewes that received a live commercial vaccine and the negative control ewes that received a sham Tris-buffer inoculation were used in the current study. In summary, ten ewes were vaccinated 5 months prior to mating with Enzovax (MSD Animal Health), the commercial vaccine used in Ireland, while the nine were given a sham inoculation of Tris-buffer (control ewes). All the ewes were challenged by subcutaneous inoculation at day 90 of gestation with 10^6^ IFU of *C. abortus* isolate C95/27*.* All sera were tested at six time-points: 1 = pre-vaccination, 2 = 14 days post vaccination, 3 = 35 days post vaccination, 4 = pre-challenge, 5 = 35 days post challenge and 6 = three weeks post lambing. All sera were thawed at room temperature while the kit reagents were brought to room temperature. The ELISAs were carried out according to manufacturers’ instructions. The format of each ELISA tested was an indirect ELISA. The S/P% was calculated for each sample to determine the titres of antibodies using the formula [OD_sample_-OD _negative control_]/ [OD_positive control_-OD_negative control_] × 100. For the LSI kit, a titre >25 S/P% was considered positive. For the IDvet kit, an S/P% ≤50% was considered negative, an S/P% greater than 50% and less than 60% was considered doubtful and an S/P%≥60% was considered positive. For the MVD-Enfer kit, an S/P% ≤20% was considered negative, an S/P% greater than 20% and less than 30% was considered doubtful and an S/P% ≥30%was considered positive.

### Statistical analysis

Statistical analysis of results was conducted on GraphPad Prism 7.03. Parametric unpaired t tests were carried out. Sensitivities (Se) of the kits were calculated using the results from three weeks post lambing and the following formula:

$$ \mathrm{Se}=\frac{\mathrm{The}\ \mathrm{number}\ \mathrm{of}\ \mathrm{diseased}\ \mathrm{animals}\ \mathrm{testing}\ \mathrm{positive}}{\mathrm{The}\ \mathrm{total}\ \mathrm{number}\ \mathrm{of}\ \mathrm{diseased}\ \mathrm{animals}} $$

The agreement between the ELISAs was determined from the Kappa coefficient (κ) and percentage concordance for samples from all time points [14] using EpiTools epidemiological calculators [15]. Concordance was calculated as the sum of positive –positive values and negative-negative values expressed as a percentage of the total number of serum samples [16].

## Results

**Fig. 1:**
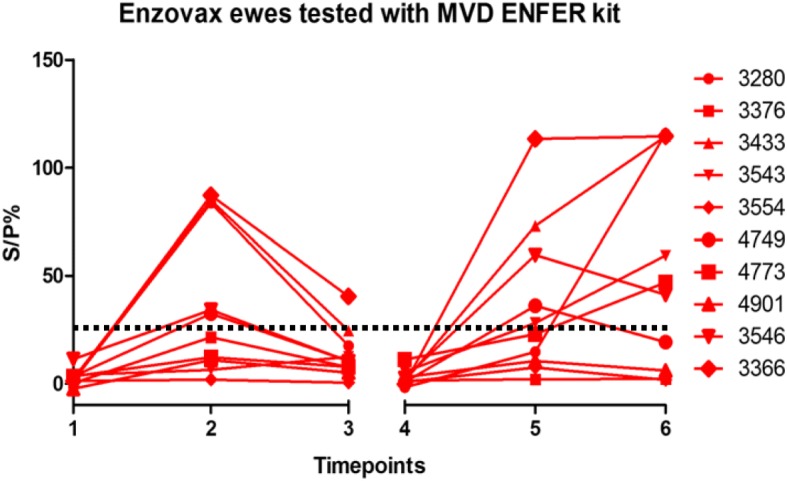
Enzovax ewes tested with MVD-Enfer ki. MVD-Enfer kit showing antibody levels in ewes vaccinated with Enzovax across the six timepoints; 1 = pre-vaccination, 2 = 14 days post vaccination, 3 = 35 days post vaccination, 4 = pre-challenge, 5 = 35 days post challenge and 6 = 3 weeks post lambing. Ewes were vaccinated on day 0 and subsequently challenged at day 90 of gestation with 1 × 10^6^ IFU of *C. abortus* C95/27. Lambing occurred from day 125 of gestation until day 145 gestation. Dashed line indicates point of sero-positivity > 30 S/P%

**Fig. 2:**
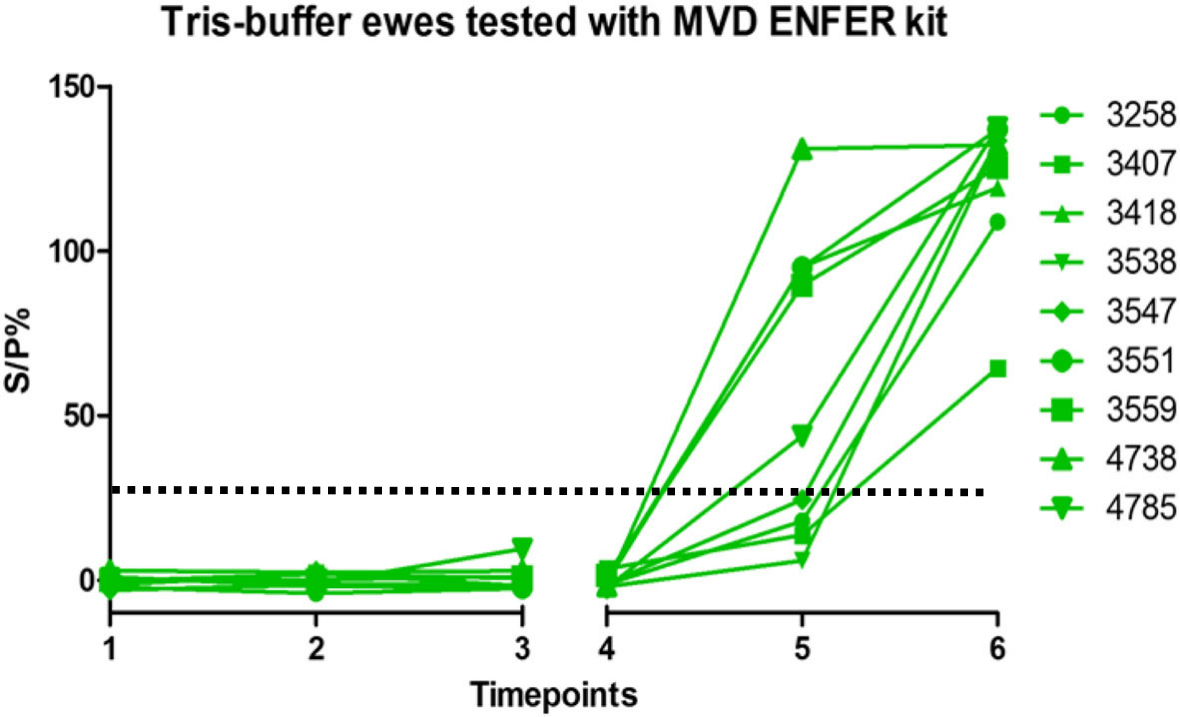
Tris-buffer ewes tested with MVD-Enfer kit. MVD-Enfer kit showing antibody levels in ewes vaccinated with Tris-buffer across the six timepoints; as described in Fig. 1. Vaccination, challenge and lambing of ewes occurred as described in Fig. 1. Dashed line indicates point of sero-positivity > 30 S/P%

**Fig. 3:**
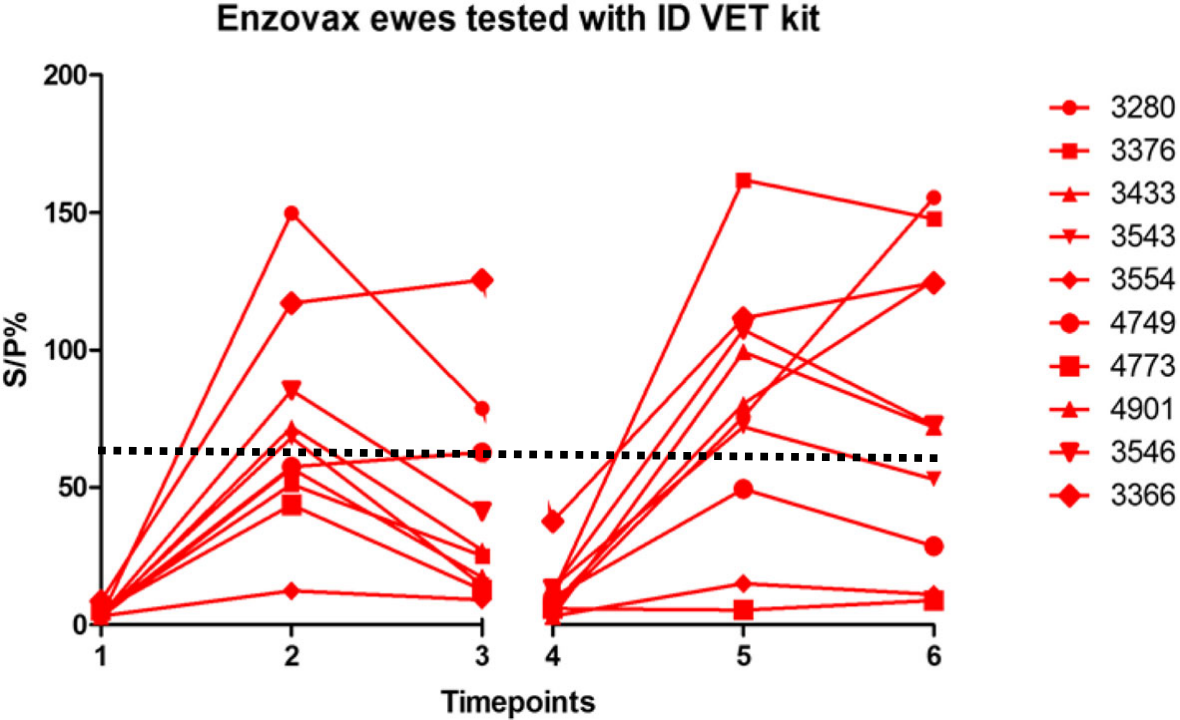
Enzovax ewes tested with IDvet kit. IDvet kit showing antibody levels in ewes vaccinated with Enzovax across the six timepoints; as described in Fig. 1. Vaccination, challenge and lambing of ewes occurred as described in Fig. 1. Dashed line indicates point of sero-positivity > 60 S/P%

**Fig. 4:**
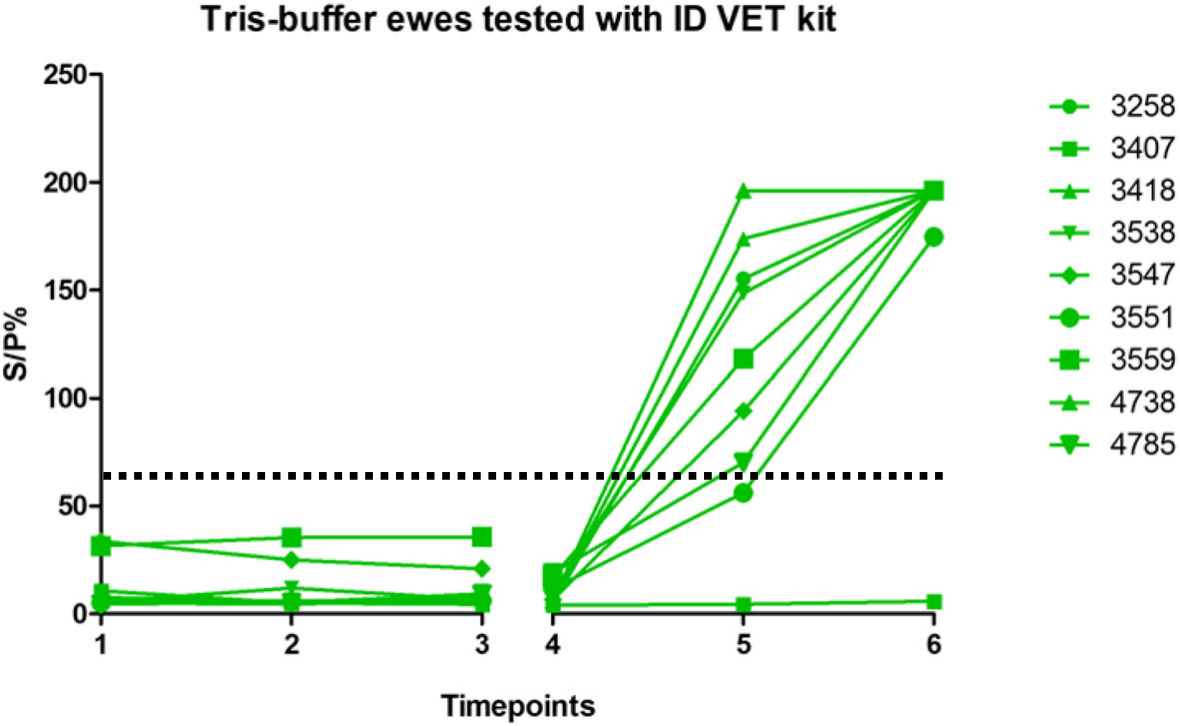
Tris-buffer ewes tested with IDvet kit. IDvet kit showing antibody levels in ewes vaccinated with Tris-buffer across the six timepoints; as described in Fig. 1. Vaccination, challenge and lambing of ewes occurred as described in Fig. 1. Dashed line indicates point of sero-positivity > 60 S/P%

**Fig. 5:**
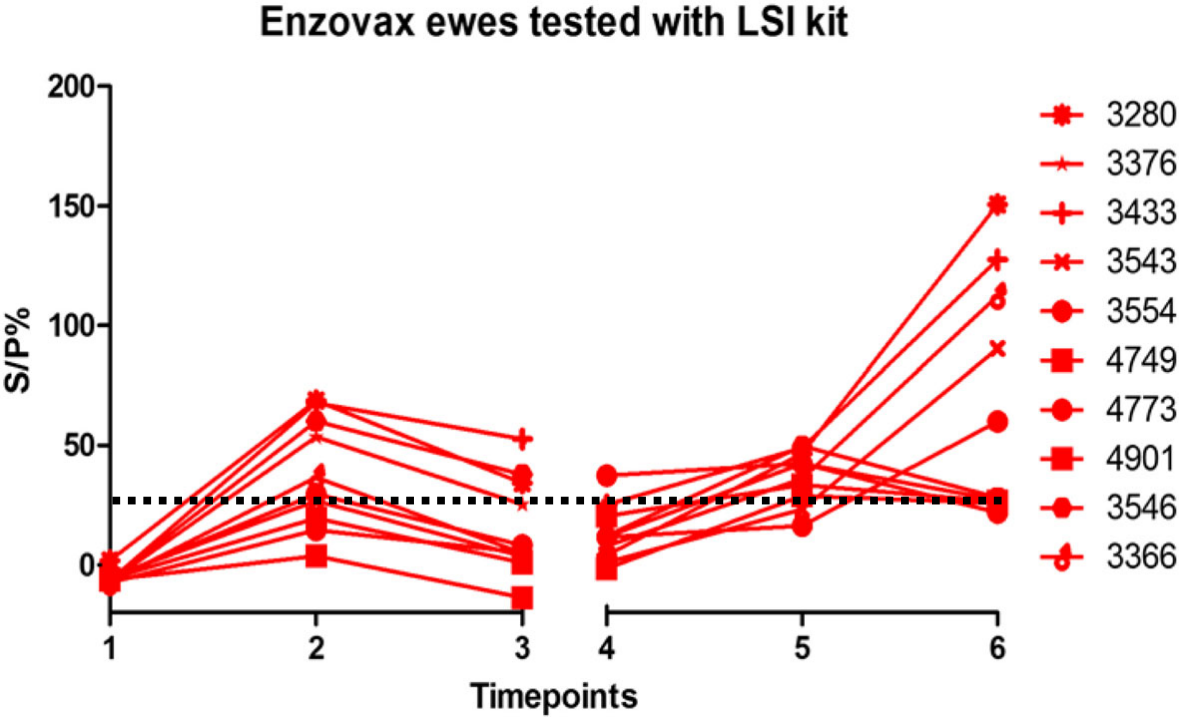
Enzovax ewes tested with LSI kit**.** LSI kit showing antibody levels in ewes vaccinated with Enzovax across the six timepoints; as described in Fig. 1. Vaccination, challenge and lambing of ewes occurred as described in Fig. 1. Dashed line indicates point of sero-positivity > 25 S/P%

**Fig. 6:**
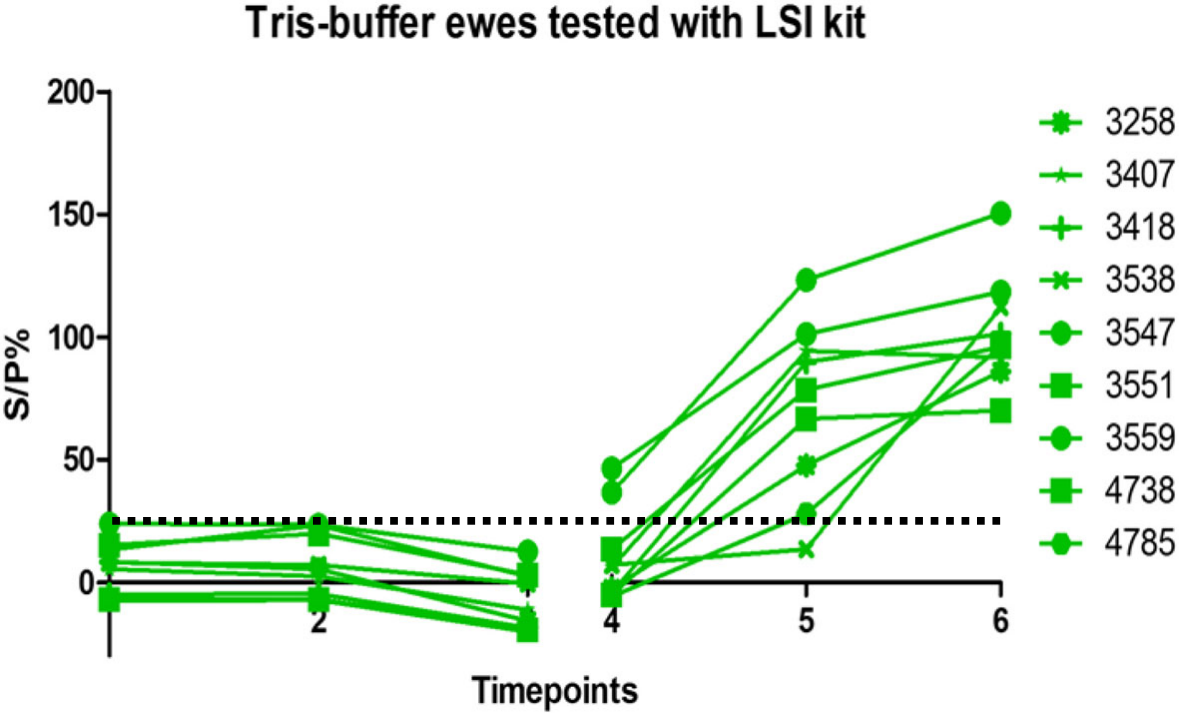
Tris-buffer ewes tested with LSI kit. LSI kit showing antibody levels in ewes vaccinated with Tris-buffer across the six timepoints; as described in Fig. 1. Vaccination, challenge and lambing of ewes occurred as described in Fig. 1. Dashed line indicates point of sero-positivity > 25 S/P%

All ELISA kits detected a similar trend in antibody levels post-vaccination and post-challenge. The antibody levels in the two groups of ewes at the six time-points are displayed in Figs. 1, 2, 3, 4, 5 and 6. The ewes vaccinated with the commercial vaccine showed a rise in antibody titre after vaccination (Figs. 1, 3 and 5). This is to be expected as it is a live vaccine and the animals were therefore exposed to the full range of chlamydial antigens. Antibody levels in both groups rose after challenge at day 90 of gestation (Figs. 1, 2, 3, 4, 5 and 6).

All ewes showed a steady rise in antibody levels after challenge, however the Enzovax vaccinated ewes at day 35 post challenge appeared to plateau (Figs. 1 and 3) unlike Tris-buffer vaccinated ewes (Figs. 2 and 4). It is important to note that in general the IDvet kit recorded higher antibody levels for both vaccine groups and peaked at levels higher than 150 S/P% (Fig. 3). The other ELISA kits did not exceed 150 S/P% (Figs. 1 and 5).

All Enzovax ewes after vaccination demonstrated a peak similar to that post-challenge, showing the difficulty of differentiating vaccinated ewes from infected ewes using the current commercial vaccine and available ELISAs.

The results of the MVD-Enfer kit for each individual Enzovax vaccinated ewe are detailed in Fig. 1. At day 14 post vaccination, four ewes tested negative and one ewe tested doubtful. Ewe number 3376 tested doubtful with an S/P% of 26.69. Ewes 3543, 3554, 4773, 4901 all tested negative, with an S/P% of 6.39, 2.56, 12.23 and 15.66 respectively. These five ewes all tested negative at day 35 post vaccination. Three ewes tested negative at both 35 days post challenge and 3 weeks post lambing, so antibody levels of these ewes did not show an increase following challenge. Ewes 3376, 3554 and 4901 had an S/P% of 1.18, 1.34 and 6.22 respectively, at 3 weeks post lambing. For the other ewes there was an increase in antibody levels after vaccination and again after challenge at day 90 of gestation.

The results of the MVD-Enfer kit for each individual Tris-buffer ewe are shown in Fig. 2. As expected, all ewes tested negative following the sham inoculation. At 35 days post challenge some ewes begin to test positive, with all ewes in the group testing positive at 3 weeks post-lambing. Ewe 3547 tested doubtful at 35 days post challenge with an S/P% of 24.48, but this ewe subsequently tested positive at 3 weeks post lambing.

The antibody levels in individual ewes from the Enzovax group using the IDvet kit are shown in Fig. 3. In general, antibody levels rose following vaccination. However, four ewes did not test positive at either 14 or 35 days post vaccination. Ewe 3376 tested doubtful at day 14 with an S/P% of 51.17 and tested negative at day 35 with an S/P% of 25.19. Ewe 3554 tested negative at day 14 and day 35, with an S/P% of 23.33 and 19.18 respectively. Ewe 4773 also tested negative at day 14 and day 35, with an S/P% of 49.81 and 25.21 respectively. Ewe 4901 tested doubtful at day 14 with an S/P% of 56.90 and tested negative at day 35 with an S/P% of 17.31. Following challenge, there was a general rise in antibody levels, although four ewes did not fit this trend. At 3 weeks post lambing, ewe 3543 tested doubtful, with an S/P% of 53.01. Furthermore, ewes 3554, 4749 and 4773 all tested negative at 3 weeks post lambing, with an S/P% of 9.04, 25.49 and 8.29 respectively.

The results of the IDvet kit for Tris-buffer ewes are shown in Fig. 4. All ewes tested negative in the vaccine phase of the study. Most ewes in the group tested positive at both 35 days post challenge and at 3 weeks post lambing. Ewe 3551 tested doubtful at 35 days post challenge with an S/P% of 56.09, but subsequently tested positive at 3 weeks post lambing. However, ewe 3407 remained negative throughout the study; this ewe did not seroconvert at either 35 days post challenge or at 3 weeks post lambing. The S/P% was 4.55 and 5.10 at these time-points respectively.

The individual antibody levels for the Enzovax ewes recorded using the LSI kit are shown in Fig. 5. In general, antibody levels increased after vaccination. However, three ewes in this group tested negative at both 14 and 35 days post vaccination. Ewes 4749, 4773 and 4901 tested negative at day 14 post vaccination, with an S/P% of 19.43, 14.62 and 3.79 respectively. These ewes also tested negative at 35 days post vaccination, with an S/P% of 0.93, 5.20 and − 13.73 respectively. As regards the challenge period, there was a general trend of rising antibody levels following inoculation. One ewe, 4773, tested negative at 3 weeks post lambing with an S/P% of 21.88, but this ewe had tested positive previously at 35 days post challenge.

Results for the Tris-buffer ewes using the LSI kit are shown in Fig. 6. All ewes tested negative in the vaccine phase. Pre-challenge, all ewes in the group tested negative apart from two ewes, 3547 and 3559. These ewes had an S/P% of 36.56 and 46.41 respectively. At 35 days post challenge, all ewes tested positive apart from one ewe, 3538, which tested negative with an S/P% of 13.40. This ewe did test positive at 3 weeks post lambing, as did all other ewes in the group.

**Fig. 7:**
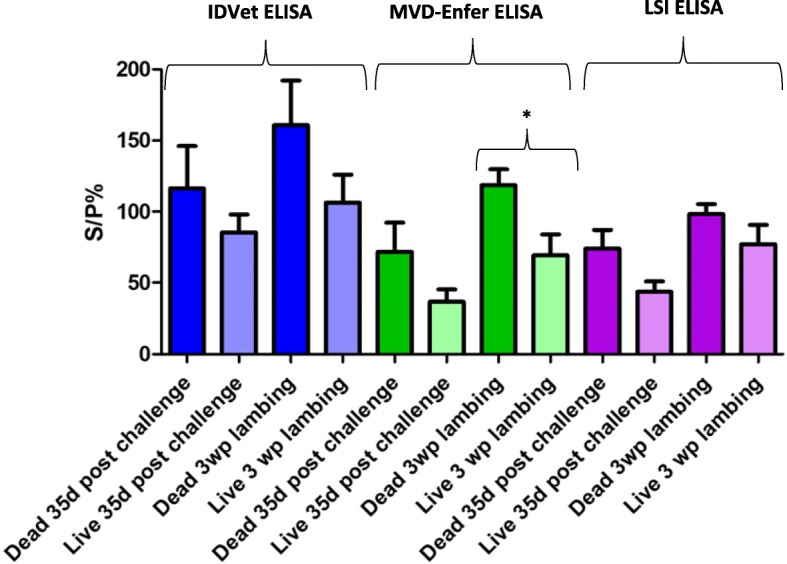
Antibody levels in ewes that had live lambs versus dead lambs. Comparison of antibody levels at 35 days post challenge and 3 weeks post lambing (~ 70 days post challenge) in ewes that had live or dead lambs. * marks a statistically significant difference between antibody levels in ewes at 3 weeks post lambing that had live or dead lambs (*P* value = 0.0482)

In an effort to determine if there was a correlation between antibody levels and whether or not a ewe would abort, graphs were constructed to examine the levels of antibodies recorded from each ELISA in ewes that aborted compared to ewes that had live lambs. All ten Enzovax vaccinated ewes had live lambs while only three out of the nine Tris-buffer control ewes had live lambs (3258, 3547 and 4785). As shown in Fig. 7, antibody levels were higher at day 35 post challenge in ewes that went on to abort compared to ewes that produced live lambs for all kits. Furthermore, antibody levels were higher again at 3 weeks post lambing than at 35 days post challenge in both ewes that aborted and ewes that had live lambs. The MVD-Enfer kit detected a statistically significant difference between antibody levels in ewes at 3 weeks post lambing that had live or dead lambs (*P* value = 0.0482). Statistically significant results were obtained with all kits with higher antibody levels at 3 weeks post lambing compared to 35 days post challenge for Tris-buffer control ewes (IDvet *P* = 0.0062, MVD-Enfer *P* = 0.0021, LSI *P* = 0.0222).

The sensitivity of each ELISA kit was calculated according to the Se equation described previously. The IDvet kit had a sensitivity of 73.68% and the MVD-Enfer kit had a sensitivity of 78.95%. The LSI kit had the highest sensitivity, at 94.74%. The kappa coefficients demonstrated that all ELISAs had a “moderate to substantial” agreement [14] with kappa values ranging from 0.59–0.70 when calculated using sera from all time points. Moderate agreement ranges from 0.41–0.60 while substantial agreement ranges from 0.61–0.80 [14]. The percentage concordance between all three ELISAs was greater than 78% as shown in Table 1.

**Table 1:** Kappa coefficient values and concordance percentages for comparing the LSI kit to MVD kits, LSI kit to ID vet kit and ID vet kit to MVD kit using all data from the 6 time points; pre-vaccination, 14 days post vaccination, 35 days post vaccination, pre-challenge, 35 days post challenge and 3 weeks post lambing

	MVD ELISA	ID VET
LSI ELISA	Kappa (κ) = 0.5887% concordance = 78.9	Kappa (κ) = 0.6842% concordance = 80.7
MVD ELISA		Kappa (κ) = 0.7012% concordance = 80.7

## Discussion

Various studies have been conducted to date evaluating the sensitivity and specificity of ELISAs compared to CFT in the diagnosis of *C. abortus* infection [9, 11, 17–20]. The results of these studies vary, with McCauley et al. [18] describing equal sensitivities and specificities between CFT and the ELISAs tested. In contrast, Longbottom et al. [11] and Markey et al. [20] reported that ELISAs are a more sensitive serological test than CFT. As knowledge of the outer membrane proteins of *C. abortus* has increased over time, new antigenic proteins have been explored and have resulted in the creation of novel ELISA plates [4, 11].

The aim of this current study was to compare three commercial ELISAs coated with three different antigens; MOMP, POMP and LPS. All ELISAs demonstrated a “moderate to substantial” agreement (0.59–0.70) [14] using the kappa coefficient test while the percentage of concordance was also greater than 78% in comparing all three ELISAs [16]. The LSI ELISA coated with chlamydial LPS was the most sensitive at 94.74%, when calculated at 3 weeks post lambing. This indicates that the LSI kit found more animals to be positive than the other kits and appears to be most effective in diagnosing the infection in individual animals. As well as sensitivity, specificity rates are essential data in determining the most effective serological test. Buendía et al. [21] suggested that mixed infections of *C. abortus* and *C. pecorum* are common in flocks. However, the specificities of these ELISAs were not calculated in this study as sera from ewes known to be infected with other chlamydial species such as *C. pecorum* were not available.

Another limitation of this study was that the small sample size with only 19 ewes included in the study. A larger sample size would be beneficial in evaluating these ELISAs and determining their sensitivities. Field sera from sheep naturally infected with *C. abortus* as well as sera from sheep known to be positive for *C. pecorum* would have been useful in evaluating these ELISAs. This would also have allowed specificity data to be calculated.

With the IDvet and MVD-Enfer kits in particular, a number of samples had to be retested as unexpected results were obtained the first time they were tested. As mentioned in the results, some ewes in the Enzovax group did not test positive at any time-point with the MVD-Enfer kit, even after retesting. This was an interesting finding and warrants further investigation.

With the IDvet kit two Enzovax ewes remained negative after vaccination and challenge, even after the samples were retested. These examples of sera that required retesting show that the accuracy of these tests must be taken into consideration and demonstrates that the results were sometimes difficult to interpret. The LSI kit therefore seemed to be the most effective kit in this study but a greater number of samples would need to be tested across more time-points to confirm this. One possible option would be to screen flocks with the LSI kit to identify all ewes infected with *Chlamydia* species and then to use the other kits to detect ewes infected with *C. abortus* only. However, this approach may not be economically feasible.

Although an LPS-based ELISA fails to distinguish between *C. abortus* and *C. pecorum* infected ewes, Griffiths et al. [22] suggest it is a more sensitive screening test than the CFT, especially in flocks where abortion has occurred. Kaltenboeck et al. [23] also showed that a chlamydial LPS ELISA was more sensitive than the CFT and may be a suitable replacement. Furthermore, a purified LPS-based ELISA prepared by Sting and Hafez [24] was shown to be more sensitive than the CFT.

As regards MOMP-based ELISAs, much of the literature has examined their sensitivities in comparison to the CFT [4, 18, 25, 26]. A competitive ELISA described by Salti-Montesanto et al. [25] diagnosed 9 out of 10 infected flocks whereas the CFT detected only 6 out of 10 infected flocks in the study. Hoelzle et al. [26] suggested that recombinant MOMP antigens are suitable for use in serological tests and may eliminate binding of cross-reactive antibodies. While McCauley et al. [18] reported a sensitivity of 70.4% when using MOMP synthetic peptide-based ELISA, other literature suggests that a MOMP ELISA based on the variable segment 2 region yields similar results to the CFT, lacks sensitivity and is therefore not suitable for use as a screening test [4].

Numerous studies have investigated the use of POMP antigens in serological assays for the detection of *C. abortus* antibodies [9, 11, 19, 21, 27]. Buendía et al. [21] reported a sensitivity of 90.9% when using an 80–90 kDa POMP-based ELISA in comparison to the CFT which was 71% sensitive, while also offering the possibility of differentiating rMOMP-vaccinated ewes from naturally infected ewes. In another study investigating a recombinant POMP80–90 ELISA a sensitivity of 80% was calculated, which was higher in comparison to the CFT [19]. However, when using field sera, a lower sensitivity was obtained [19]. Longbottom et al. [27] described an indirect ELISA created using a recombinant POMP91B fragment which yielded a sensitivity of 84.2%, which was greater than that of the CFT. The ELISA also proved more effective at distinguishing between *C. abortus* and *C. pecorum* infections [27]. A further study was carried out based on a recombinant POMP90 ELISA [11]. Both the rOMP90–3 and rOMP90–4 ELISAs had higher sensitivities than the CFT, with the rOMP90–4 fragment being more sensitive than the rOMP90–3 fragment when testing field sera [11]. In contrast, Wilson et al. [9] reported the rOMP90–3 ELISA as being more sensitive than both the CFT and the rOMP90–4 ELISA, with results of 96.8, 93.5 and 91.9% respectively. A sensitivity of 96.8% with the rOMP90–3 ELISA [9] is higher than the result of 78.95% obtained in the current study.

Interestingly anti-chlamydial antibody levels were found to be consistently higher after challenge in ewes that aborted compared to ewes that had live lambs. Studies have shown increases in antibody levels associated with *C. abortus* reproductive failure [28–30]. Gutierrez et al. [31] and García-Seco et al. [32] have also noted that antibody titres were lower in animals that did not go on to abort. This current study and others clearly show how elevated antibody levels could form a basis to identify ewes that will go on to abort. The antibody increase observed in all groups at 3 weeks post lambing could be associated with the development of protective immunity against subsequent abortions as described by García-Seco et al. [32] and Longbottom and Coulter [33]. Alternatively it may simply be a diagnostic marker of exposure to high numbers of circulating *C. abortus*. In any case early warning of impending abortions could have significant benefits in terms of targeting long- acting antimicrobial treatment [3] towards those animals in imminent danger of abortion rather than having to administer treatment to the whole flock.

It is clear from the literature available that a lot of variation exists in the sensitivities of different ELISAs. In general, ELISAs have been shown to be more sensitive than CFT, in particular the POMP ELISAs as described above. Hagemann et al. [34] described how antibodies to virulence-associated antigens such as chlamydial protease-like activity factor (CPAF) are not present for more than 18 weeks after abortion in most cases, whereas antibodies to surface antigens such as MOMP persist for longer. However, in ewes that lamb normally, antibodies to surface antigens do not persist [34]. It has also been shown by Livingstone et al. [4] that anti-MOMP antibodies only increase around the time of abortion and then gradually decline, while anti-POMP antibodies appear earlier and may persist for longer. It is therefore evident that differences in immunoreactivites may be due to the time-points at which blood samples are taken following abortion, as described by Forsbach-birk et al. [35]. It is important to note that the current study was limited to six specific time-points and therefore the peak level of antibody, after abortion may have been missed. Future work would be valuable in assessing these ELISAs using sera from different time-points, for example at various points throughout gestation and beyond 3 weeks post lambing.

## Conclusions

This study demonstrated that when sera are tested at 3 weeks post lambing the LSI kit was the most sensitive of the three commercial kits examined. As the LPS used in the LSI kit is cross-reactive with all chlamydia, it is suited to identifying flocks infected with any chlamydial species. It is clear from the data presented that ewes vaccinated with Enzovax usually become seropositive at 14 days after vaccination. This study also showed that antibody levels are higher in ewes that abort compared to ewes that have live lambs. In both ewes that aborted and ewes that have live lambs, antibody levels were higher at 3 weeks post lambing than at 35 days post challenge. Future work involving more sera, as well as testing at more time-points, would be beneficial in confirming the results obtained in this study.
